# Supplementary material for: The age bias in labeling facial expressions in children: Effects of intensity and expression
Source: PLoS One. 2022 Dec 2;17(12):e0278483. doi: 10.1371/journal.pone.0278483 (PMC9718404; doi:10.1371/journal.pone.0278483)
Supplement: S1 Appendix — (DOCX) [file pone.0278483.s001.docx]

**Appendices**

***Appendix 1***

*Procedure of the computer task, copied and adapted from Gao & Maurer, 2009*

The participant was pointed towards the screen, on which four houses were visible, each with an emoticon on it: happy, sad, disgusted and neutral. The participant was told: “In one of these houses a story is told which makes the people sad. Can you point which house that would be?”. After the participant correctly guessed the house for happiness, sadness and disgust, the researcher would say: “In one of the houses no story is being told and people are not feeling anything. Can you point that house for me?”. After the participant correctly identified the neutral house, he/she was told: “You will be seeing pictures of faces. Your job is to bring each person to the right house for them. The person can only go to a house when they are feeling the same emotion as the people in that house. If someone has to go to the sad house, you can click here [now the right key was pointed out]; If someone has to go to the disgust house, you can click here [now the right key was pointed out]; If someone has to go to the happy house, you can click here [now the right key was pointed out]; If someone has to go to the house without an emotion, you can click here [now the right key was pointed out]. It is important to know that sometimes the whole group is happy, but that some people are a little bit happy and some people are very happy. During this game, they all go to the happy house. This is also true for the people who are experiencing sadness or disgust. Thus, it is not important how much sadness, disgust or happiness they are feeling. The faces will stay on the screen until you give an answer. Before you see a face, you will see a little cross in the middle. This helps you remember to look to the middle of the screen. We are going to practice first. In the practice round you will get a reminder after every face of which emoticon belongs to which key. During the actual task, you will not be getting this reminder no more, but you can look on this sheet of paper to remember.
