# Supplementary material for: The age bias in labeling facial expressions in children: Effects of intensity and expression
Source: PLoS One. 2022 Dec 2;17(12):e0278483. doi: 10.1371/journal.pone.0278483 (PMC9718404; doi:10.1371/journal.pone.0278483)
Supplement: Attachment — Submitted filename: Response to reviewers.docx [file pone.0278483.s002.docx]

Dear Dr. Walker and reviewer,

Thank you very much for evaluating our paper, and providing the opportunity to revise the manuscript. We appreciate your suggestions for improvement. We will reply to all individual comments here and have changed the manuscript accordingly.

Reviewer #1:

Comment: Thank you for inviting me to review this manuscript. It explores an interesting area that is still new but seen a lot of research interest in the past year. I think that the MS is well written and well-presented but I would like the authors to address a few comments and concerns. I will recommend a minor revision with a potential to accept after the changes made and I re-reviewed the paper.

Response: We thank the reviewer for taking the time to thoroughly read the manuscript, for these compliments and below suggestions.

**Abstract**

Comment: If length restriction allow, the authors could elaborate the details of their study and results a bit more.

Response: We thank the reviewer for this suggestion, and have now added a more extensive abstract.

**Introduction**

Comment: Clearly written, easy to follow and justifies the research presented later.

Response: We thank the reviewer for these compliments.

**Methods**

Comment: Methods are described in sufficient detail to understand the approach used and are appropriate statistical tests applied. This is a simple, yet efficient and very clever design.

Response: We thank the reviewer for these compliments.

Comment: Regarding the sample size authors should provide an estimation justifying the number of participants included. The huge discrepancy between the number of adults and children could be an issue and a potential bias in the analyses.

Response: There is indeed a large discrepancy between the age-groups in sample size. Important to consider here is that both sample sizes are large enough to yield high power in the analyses (see also below on the app by Baker and colleagues (2020)). The reason for the relatively small sample of adults is that the main aim of this study is to investigate emotional labeling in children. The adult group was primarily added for comparison of conclusions. The reason for the relatively large sample of children is two-fold: first, we aimed for multiple children per age-year to prevent outliers within this age affecting the overall conclusions, and to be able to exploratorily investigate developmental effects within the children. Second, this study was part of a museum exhibition and we wanted to allow as many children as possible to experience taking part in a scientific study. The current sample of children is the total number that participated during the exhibition. Nevertheless, indeed the sample size affects the variation and as such the analyses. We contemplated binning the children in smaller groups (e.g. per two years) for exploratory analyses. However, although this would lead to more comparable sample sizes with the adult group, the bins would be relatively arbitrary and would remove the information from children at the border of the bin. Instead, we included age as a continuous variable in our exploratory correlation analyses. We now added a summary of this information to the participant section on page 6 and to the limitation section on page 20.

Comment: I might have missed it (in which case, please forgive me), but why did you only use disgust, sad an happy expressions? There is no neutral control. What about fearful or angry faces?

Response: In this study, we combined the stimulus presentations used by Griffiths and colleagues (2015; 2017) and by Gao and Maurer (2009). The study by Gao and Maurer was of particular interest for selecting expressions that children might not yet label accurately and what this expression is confused with. That study showed that particularly sad faces are difficult to label, and are confused with neutral or disgust. For this reason, we included sad and disgust expressions in the current study. On the other hand, even the youngest children could label happy expressions as well as adults, even for the lowest intensities. As such, we included happy faces as a proof of principle for our experiment. We added a neutral control in the form of the 0% intense faces, which were the neutral expressions in the original dataset.

Originally, we also included fearful faces, because Gao and Maurer (2009) showed that labeling of this expression develops between 5 and 7 years of age, and is in all children confused with surprise. Moreover, fearful faces were of interest because of the fear-bias often reported in infancy and even adulthood (add refs). However, after a pilot study it appeared that including four expressions made the experiment lasting too long for the youngest children to participate. As such, we decided to remove fearful expressions from the experiment. Based on Gao and Maurer (2009), we did not include angry faces because this was labeled correctly at 100% intensity and confused only with neutral (not another expression) at lower intensities in all children. Thus, we expected less of a developmental effect for labeling of angry than sad expressions. We now include a summary of this reasoning in the methods on page 8.

Comment: Were there any steps taken to control for the low-level features of the pictures either before the experiment or after that? Such as using an algorithm to equate them on contrast, spatial frequency, brightness, etc. OR using these parameters as covariates in the analysis. There might be a difference between picture categories on these variables. I do not mean the distortions resulting from the morphing process.

Response: Indeed, low-level features are very important in the processing and labeling of emotional faces, as sensitivity to several features continues to develop throughout childhood (van den Boomen et al., 2012), and such features are used differently in different age-groups for processing emotional expressions (e.g. Jessen and Grossman, 2017; Peters et al., 2017; van den Boomen et al., 2019; Vlamings et al., 2010. However, as the focus of the current study was the labeling of the emotional content of faces with different emotional expressions, our main criterion for choosing stimuli out of a validated Radboud stimulus database was the percentage of agreement on the emotional label amongst responders in the validation study. As such, we choose this percentage instead of the equality on low-level visual properties. Furthermore, as we aimed to use a task that is realistic to administer in young children, we used a similar task as Gao and colleagues (2009) and Griffith et al (2015) with the exception that we used four instead of 2 trials per condition. Due to this relatively low number of trials, there is quite some variation in low-level visual properties between stimuli. However, there are too many potential properties to realistically include as covariate in each of the analyses, because for instance within spatial frequency we would need to include multiple bins of lower, middle, and higher spatial frequencies. As we agree that low-level visual properties could play a role in the reported effects, we have now added a summary of this to the limitation section on page 19.

Comment: Speaking of which, the authors write that “distortions resulting from the morphing process were fixed with Photoshop”. How does this change the parameters of the pictures? Could this process be considered as something that made the pictures be more alike in terms of low-level visual features?

Response: Most observed distortions were parts of the eyes and mouth that were overlapping between being close and open. For example, if the 100% image had an open mouth and the neutral face a closed mouth, some intermediate intensities had strange-looking parts of the mouth where both the teeth and the overlapping skin was visible. This was corrected by creating a partly-opened mouth, appropriate for the intensity. By replacing the distortion with a more naturally-looking part of the mouth or eye, we likely included more higher and fewer lower spatial frequency, as the clearer borders contain higher and the blurry overlap lower spatial frequencies. Similarly, we likely created higher contrast images. This made the different intensities likely more alike in terms of low-level visual features. As similar distortions were observed amongst actors, and corrected in a similar way, this likely didn’t affect the comparison of low-level visual features between actors. We have now included this potential effect to the limitation section on page 19.

Comment: How did you decide what trial count to use? Was this considered as part of a power analysis? In other terms, is the variance in performance measured adequately in this study? There is a recent work by Baker and colleagues (2020) who addressed this issue and even offered a shiny app to calculate sample sizes: <https://shiny.york.ac.uk/powercontours/>

Response: Previous studies in this age-group that investigated emotional labeling in different intensities (Gao et al., 2009) or stimulus age (Griffith et al., 2015) used two trials per condition. This study aimed to apply the methods used by Gao et al. (2009) as close as possible, with the main difference that we digitized the experiment. However, as we thought two trials per condition is quite low, we decided to include four trials per condition to allow for a bit more variation in performance. We understand and agree with the concern that the possible variance within each participant is still rather limited, as this allows for conclusions on a limited optional outcome in sensitivity. Potentially related to this limited options for outcome per participant, the distribution of outcomes between participants was not normal. Consequently, we applied non-parametric analyses that do not assume such normal distribution. Moreover, based on the power calculation using the app by Baker and colleagues (2008) the current study still has a high power, even with the four trials and for the smaller adult sample. We now added the limited variance, its effect on the possible sensitivity outcomes, and the power information to the discussion on page 20.

**Results**

Comment: Maybe use the symbol tau instead of letter t.

Response: We now use the symbol tau.

**Discussion**

Comment: The conclusions a reasonable extension of the results. Please state the strengths and weaknesses or limitations of your study clearly.

Response: We thank the reviewer for this compliment and suggestion, and have added a paragraph on strengths and weaknesses on page 19 and 20.

Comment: The current study is focusing on labeling but how does this relate to detection? Please discuss your results in the light of these papers.

Tamm, G., Kreegipuu, K., Harro, J., & Cowan, N. (2017). Updating schematic emotional facial expressions in working memory: Response bias and sensitivity. Acta psychologica, 172, 10-18.

Zsido AN, Arato N, Ihasz V, Basler J, Matuz-Budai T, Inhof O, Schacht A, Labadi B and Coelho CM (2021) “Finding an Emotional Face” Revisited: Differences in Own-Age Bias and the Happiness Superiority Effect in Children and Young Adults. Front. Psychol. 12:580565. doi: 10.3389/fpsyg.2021.580565

Response: We thank the reviewer for pointing to these interesting papers and this discussion point. We have now included an elaborate discussion on page 17 and 18, focusing on the different components of emotion reasoning (including detection and labeling) and processes underlying these components (including working memory). In this discussion, we also explored whether biases discussed in these suggested papers can explain the reported biases in the current manuscript.
